# Reappraisal of clinical trauma trials: the critical impact of anthropometric parameters on fracture gap micro-mechanics—observations from a simulation-based study

**DOI:** 10.1038/s41598-023-47910-2

**Published:** 2023-11-22

**Authors:** Michael Roland, Stefan Diebels, Marcel Orth, Tim Pohlemann, Bertil Bouillon, Thorsten Tjardes

**Affiliations:** 1 Chair of Applied Mechanics, Saarland University, Campus A4 2, 1. OG, 66123 Saarbrücken, Germany; 2 Department of Trauma, Hand and Reconstructive Surgery, Saarland University, Kirrberger Strasse 100, 66421 Homburg, Germany; 3 Chair for Orthopedic Surgery, Trauma Surgery and Sportstraumatology, Department of Orthopedic Surgery, Trauma Surgery and Sportstraumatology, Cologne Merheim Medical Center, University Witten/Herdecke, Ostmerheimerstrasse 200, 51109 Cologne, Germany

**Keywords:** Preclinical research, Biomedical engineering

## Abstract

The evidence base of surgical fracture care is extremely sparse with only few sound RCTs available. It is hypothesized that anthropometric factors relevantly influence mechanical conditions in the fracture gap, thereby interfering with the mechanoinduction of fracture healing. Development of a finite element model of a tibia fracture, which is the basis of an in silico population (n = 300) by systematic variation of anthropometric parameters. Simulations of the stance phase and correlation between anthropometric parameters and the mechanical stimulus in the fracture gap. Analysis of the influence of anthropometric parameters on statistical dispersion between in silico trial cohorts with respect to the probability to generate two, with respect to anthropometric parameters statistically different trial cohorts, given the same power assumptions. The mechanical impact in the fracture gap correlates with anthropometric parameters; confirming the hypothesis that anthropometric factors are a relevant entity. On a cohort level simulation of a fracture trial showed that given an adequate power the principle of randomization successfully levels out the impact of anthropometric factors. From a clinical perspective these group sizes are difficult to achieve, especially when considering that the trials takes advantage of a „laboratory approach “, i.e. the fracture type has not been varied, such that in real world trials the cohort size have to be even larger to level out the different configurations of fractures gaps. Anthropometric parameters have a significant impact on the fracture gap mechanics. The cohort sizes necessary to level out this effect are difficult or unrealistic to achieve in RCTs, which is the reason for sparse evidence in orthotrauma. New approaches to clinical trials taking advantage of modelling and simulation techniques need to be developed and explored.

## Introduction

Fractures of bones are a global burden. Surgical fracture care improved the results dramatically over the last 60 years in respect to anatomical healing and restoration of function, but a wide availability is still limited to an advanced health care system. Even with all progress in minimizing perioperative surgical trauma and precision in planning and execution of a surgical stabilization, complications like infection, delayed healing and non-healing (non-unions) can compromised the expected result dramatically. Therefore, primary fracture healing and fracture non-union are the opposing ends of a continuum^1–3^, rather than precisely defined entities of their own, with an estimated 10% non-union rate after surgically treated fractures. This complication exponentially increases the individual burden of disease by multiplying treatment time and surgical interventions and additionally challenging the healthcare system by dramatically rising treatment costs, calculated for a tibial non-union with 25.500,- US$ in the USA^4^ and a range between 8.000€ and 91.000€ in the UK^5,6^.

Fracture healing is a process designed to completely restore shape and function of a destroyed anatomical structure by a complex, self-organising multi-scale process. The impact of mechanical stimuli on bone physiology, remodelling and repair was first described by Wolff’s law^7^, with Perren’s strain theory^8,9^ and Frost’s concept of the *“mechanostat”*^10,11^ providing the conceptual foundations for today’s surgical fracture care. Consequently, mechanical and implant related aspects of fracture care dominated research and development for a considerable period of time^12^. In 2007 Giannoudis and colleagues^13^ conceptualized fracture healing as a multi-scale and multi domain process such that the biological aspects of fracture healing moved into the focus of clinical and laboratory research^2^.

However, neither the conceptual developments named above, nor the fact that there is persuasive evidence from basic science regarding the induction of cellular processes in osteocytes following mechanical stimuli^14^, translated into randomized clinical trials to provide convincing evidence on efficacy and efficiency of surgical fracture care. On the contrary, a Cochrane analysis starting with 910 trials on surgical therapy of distal tibia fractures in adults^15^ finally included only three trials that proved compliant with the methodological requirements of the Cochrane Collaboration.

This fact indicates a structural problem in conducting valid clinical trials, as the unplannable trauma origin of the fracture implicates an extremely wide range of fracture mechanisms, fracture pattern, soft tissue involvement as well as nearby uncontrollable additional patient related factors as age, concomitant diseases, physical status and finally personally motivation. Even with a detailed definition of the inclusion criteria of the study groups, a clinical study will either lack the required number of included, comparable fractures for comparison or has to compromise strict inclusion criteria. Therefore, now new strategies are introduced to explore possible, up to know unknown additional factors influencing uneventful bone healing.

Given the absence of clinical evidence, and accepting the fundamental notion that mechanical^16^ as well as biological factors^13^ are the key players of fracture healing, the question arises whether there are hidden factors affecting the process of fracture consolidation, which might have been (systematically) disregarded so far. We hypothesise that the disregard of mechanically relevant anthropometric factors, which have never been considered as independent factors in clinical trials, by way of their natural variance introduce bias to an extent such that the mechanical conditions in the fracture gap vary to a degree that fracture healing as a primary outcome parameter of clinical trials cannot be realistically assessed.

## Methods

Three different scales, anthropometric measures, i.e., length of the tibia, body height and weight (macro-scale), the fracture gap (meso-scale) and the mechanics inside the fracture gap (micro-scale) need to be addressed simultaneously and consistently. There is no evidence on anthropometric parameters in conjunction with fracture healing trials. Thus, retrospective cumulative approaches like meta analyses are not suitable. The notion that anthropometric parameters interfere with fracture healing and therefore need to be considered when stratifying the cohorts of clinical trials is straight forward. Yet the actual effect anthropometric parameters excerpt, neither quantitatively nor qualitatively, is naturally unknown to date. Hence it is not possible to simply add these parameters to the set of parameters the trial cohorts should be normally distributed for, without any information on the degree and extend mechanoinductive processes might be influenced. Given the current state of knowledge this would mean to introduce another bias into a system subjected to too many biases to produce clinically meaningful results. Theoretically information on the effect of anthropometric parameters can be gathered from systematically initiated trials, or alternatively, from large multi modal registries—both approaches, although methodologically sound, suffer from logistical, epidemiological, funding and ethical impracticability. At the current stage of the investigation the mechanical implications of fracture healing are in the focus of interest. The recent developments of image processing, simulation and clinical biomechanics offer tools that allow for the controlled simulation of the mechanical component of the fracture healing process. Which is the first to be analysed, before the biological components can be investigated. At the same time the toolbox of biomechanical simulation and image processing provides techniques to create custom made in silico cohorts. Thus, in silico modelling is the method that effectively allows to address the mechanical and epidemiological issues described above.

In a three-step bottom up process, a model is developed that comprises these scales such that the effects of anthropometric parameters can be mapped to the micro-scale of the fracture gap. Varying these parameters allows to generate a population of avatars. Mimicking the recruitment process of a clinical trial, cohorts can be randomly selected from the population of avatars.

### Modelling the micromechanics inside the fracture gap: specification of mechanically relevant parameters

The mechanobiological concept of fracture healing based on mechanical stimulus and interfragmentary movement is adopted according to Carter et al.^17^ and Claes et al.^18^.

**Figure 1:**
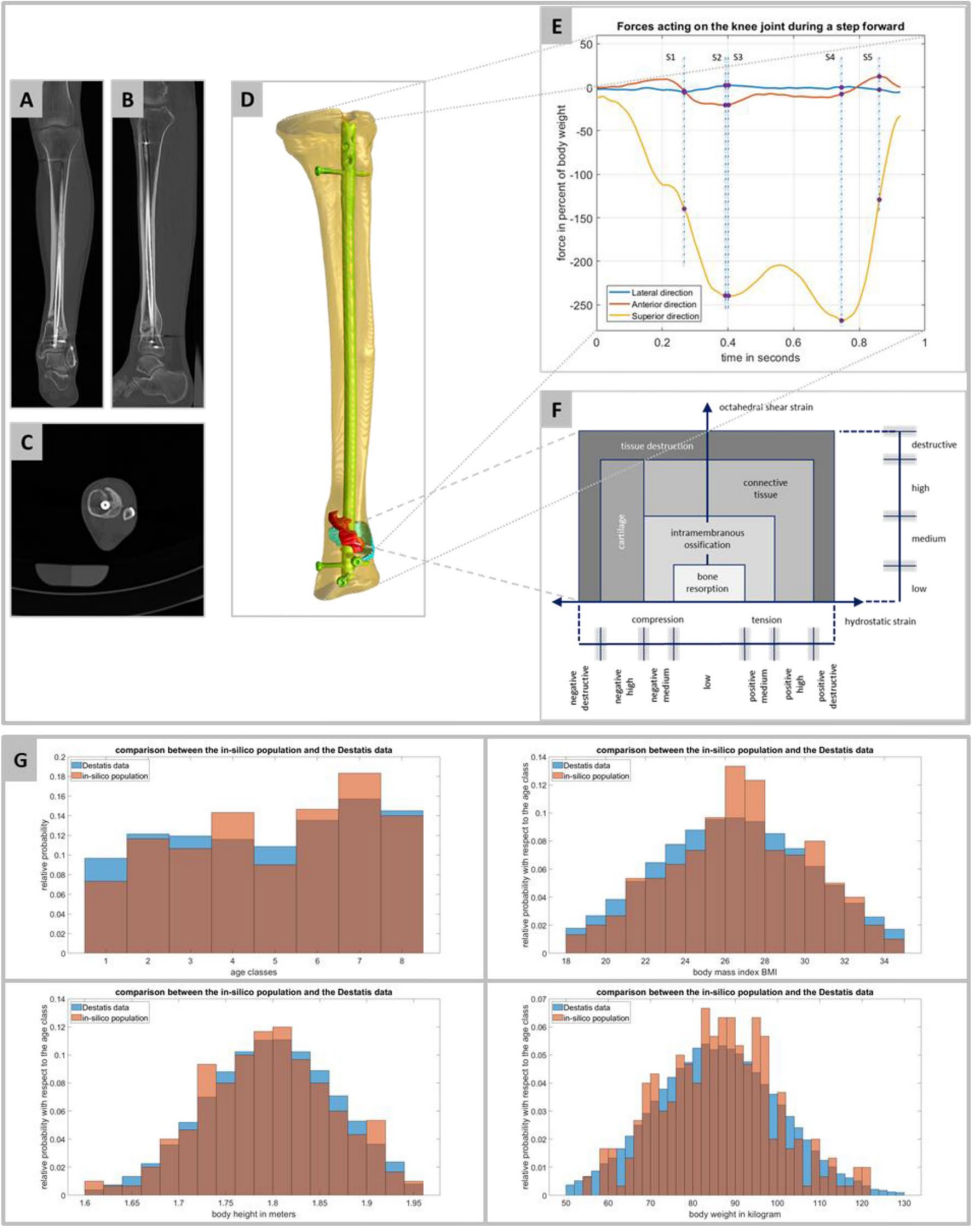
Illustration of the simulation concept and the underlying in silico population: (**A**) and (**B**) are showing the coronal and the sagittal representation; (**C**) is one slice of the image stack in axial direction showing the two-rod calibration phantom; (**D**) shows the geometrical model of avatar 0, the result of the image processing steps; (**E**) illustrates the forces acting on the knee joint during a step forward. The data is taken from the OrthoLoad database referenced to the patient with the ID “K8L”^34^. The points in time S1 to S5 are the selected landmarks for the simulation workflow. (**F**) Shows one model for the mechanobiological regulations based on mechanical quantities^19^; for each FE simulation, the relevant strain quantities for each FE tetrahedral mesh cell were evaluated and assigned to the plane shown here, adopted from^19^, in analogy to Braun and colleagues^35^ and Orth and colleagues^36^ (**G**) Comparison between the generated in silico population and the Destatis Microcensus 2017 data for Germany. The generated trial cohort is within the expected range for all parameters and describes the addressed population group quite accurately.

*Mechanically* this translates to the interplay of local volume change and local shape distortion, described by two invariants of the strain tensor, namely the volumetric strain and the octahedral shear strain derived from the deviatoric part of the strain tensor (Fig. 1F)^19,20^.

*Biologically* the experimental work of Bishop et al.^21^ demonstrated that the level of mechanical deviatoric strains is the main determinant of cell differentiation resulting in tissue formation^22–24^. Garcia et al.^22^ and Doblare et al.^24^ identified J2, i.e. the second invariant of the deviatoric strain tensor, as key quantity to describe the influence of strain on fracture healing.

*Technically* the meso-scale dimensionality of voxels in computed tomography (CT) data is transferred to a Finite Element (FE) model which constitutes as the missing link between the mechanically-driven macro-scale environment resulting from weight bearing and the biology-driven micro-scale environment at the level of osteogenic cells, which is necessary to model the impact of weight bearing and anthropometric variability on micromechanical environment in the fracture gap. Each mesh element in the fracture gap is considered a virtual meso-scale lab of fracture healing, sufficiently small to assume that the observations from *in-vitro* experiments of fracture healing can be applied, and sufficiently large that a finite number of elements fill the fracture gap.

### Finite element model of the fracture and the fractured bone, i.e. avatar 0

A tibia fracture (AO 43 A1^25^, male, 32 years, bodyweight 79·5 kg, body height 179 cm, tibia length 397 mm, body mass index (BMI) 24·81) treated with reamed intramedullary nailing (DePuy, ACE cannulated, straight working section tibia nail, 11 mm/375 mm, single screw fixation proximal, triple screw fixation distal, 8° Herzog bend 61 mm from nail tip, 2° bullet style tip) serves as blueprint for the virtual cohorts developed in the upstream steps. Full weight bearing was achieved four months after surgery (Fig. 1A–C). CT images (Somatom Definition Flash, Siemens, Germany, standard calibration phantom^26^) were used to generate the FE model. The image stack consists of 1,322 square frames with 512 pixels as width and height. The pixel spacing is 0.44 mm and the distance between two images, i.e., the voxel spacing, is 0.30 mm. The CT acquisition was performed using the following specifications: slice thickness 0.75 mm, tube voltage 120 kVp and tube current 87 mA.

Image processing with Simpleware ScanIP (Synopsys, Mountain View, CA, USA) followed a five-step workflow:

i. Segmentation of four masks: (a) intramedullary nail, (b) bone, (c) fracture gap, i.e., the space between the cortical edges of the fractured bone, and (d) callus area, i.e., newly formed bone around but not in the fracture gap (Fig. 1D) via adaptive thresholding w.r.t the calibration phantom, supplemented by a morphological close filter with isotropic values (two pixels in every spatial direction) for the fracture gap and the callus area.
ii. Mask smoothing (recursive anisotropic Gaussian filter) with the following values: fracture gap and callus area mask (one pixel in x- and y-direction, i.e., the image plane and two pixels in the z-direction), intramedullary nail and the bone mask (two pixels in x- and y-direction, and three pixels in the z-direction).
iii. Island removal for each mask combined with a cavity fill and a fill gaps procedure with priority order.
iv. Visual control of segmentation results (TT) to ensure that all physiologically and mechanically relevant areas of the fracture and the newly formed bone are appropriately mapped (Fig. 1D).
v. Generation of the FE meshes for the simulation (Abaqus, Dassault Systèmes, Velizy-Villacoublay, France) using quadratic finite elements (C3D10, ten-node tetrahedral element with four integration points) with adaptive mesh resolution for each mask. Homogenous material parameters for the implant (medical titanium alloy^27^), the fracture gap and callus area (both modelled as initially connective tissue^18^) were taken from the literature.
vi. Homogeneous material parameters based on the Hounsfield units with respect to the calibration phantom were chosen for each, cortical and trabecular bone^28,29^. Thus, the method demonstrated by Trabelsi and colleagues^30^ for the femur was adapted for use on the tibia. After calibrating the grayscale values, the density-modulus relationship for the tibia given by Rho and colleagues^31^ was used to assign every mesh cell with an isotropic material model:
  $$E = \mathrm{6,570}\cdot {\varrho }_{app}^{1.37}$$

This sequence was performed on the real clinical image data, partly manually, partly automated, and reviewed by the authors after each step. After that, the workflow could be automated via scripting in the Simpleware ScanIP software for the generation of the n = 300 avatars.

### *Generation of an *in silico* population of avatars (n* = *300)*

The in silico population is restricted to men aged 20–60 years, body height 1·60–1·95 m and BMI 18 to 35. This excludes underweight and adiposity due to their bias on fracture healing as well as geriatric, paediatric or adolescent aspects of bone physiology. Thus, downscale homogeneity (adherence to comparable mechanisms and dynamics at the micro-scale) of the model is given. The ensuing generic cohort mirrors the statistical dispersion of the corresponding general population (*Microcensus 2017—Health Questions* of the German Federal Statistical Office, Destatis). The cohort is separated into age groups (five-year increments) using the *randsrc* function (Matlab R2021b environment, Mathworks Inc., USA). Technically, means and standard distributions of the data given by the *Microcensus 2017* for each age group are mapped with the *makedist* function and truncated to the chosen restrictions with the *truncate* function. Then the random number generator *random* assigns body height and BMI independently. Body weights of the avatars are derived from body height and BMI. Figure 1G shows a comparison of the in silico cohort and the target population. Tibia lengths are derived from body height and age group with respect to the corresponding standard deviation based on forensic medicine formulae^32^, and stored in the avatars meta data. A principal component analysis of the anatomical shape of the tibia showed that variation of the tibia length accounted for 96 percent of the tibial shape variation^33^ thus technically the variation of body height, is achieved by adapting the image stack of avatar 0 in the diaphyseal segment directly to each of the given tibia lengths by inserting or deleting stack layers.

### Simulation of the stance phase of the gait cycle

Force maxima of the three spatial directions according to the OrthoLoad database^34^ (patient “K8L”, male, body weight 755N) serve as base line values and landmarks of gait kinematics for the simulation describing the full range of forces occurring in the stance phase (Figs. 1E, 3A). Rescaling these landmarks S1-S5 adjusts the avatars to the corresponding body weights matching the statistical dispersion of the target population via the boundary conditions of the FE simulations.

### Correlation between anthropometric parameters and the mechanical stimulus in the fracture gap

After running the FE simulations at S1 to S5, for each of the n = 300 avatars (total of 1500 simulations), the mechanically relevant strain data is evaluated for all 28,689 mesh cells of the fracture gap and all 47,129 mesh cells inside the callus area. Based on the strain tensor, the mechanical quantities (hydrostatic strain, octahedral shear strain, max principal strain, J2) were computed in each mesh cell and stored as data vectors. Afterwards, these 1500 data vectors are grouped in accordance with the five landmarks. These data vectors are subjected to a descriptive statistical analysis (SPSS Statistics 27, IBM Corp, Armonk, NY, United States) together with the anthropometric factors of the associated avatars. The correlation between anthropometric parameters and the local mechanical stimulus, is analysed by means of a two-sided Pearson correlation test for S1 to S5 of the stance phase. Therefore, the descriptive statistical values mean, median, interquartile range, median absolute deviation and the 95th, 90th percentiles are evaluated for the different identified quantities describing the mechanical stimulus and the interfragmentary movement for all 1500 FE results. In the results section, we restrict ourselves to the results concerning the median of the mechanical quantities, since the other statistical values yield similar results.

### *Influence of anthropometric parameters on statistical dispersion between *in silico* trial cohorts*

**Figure 2:**
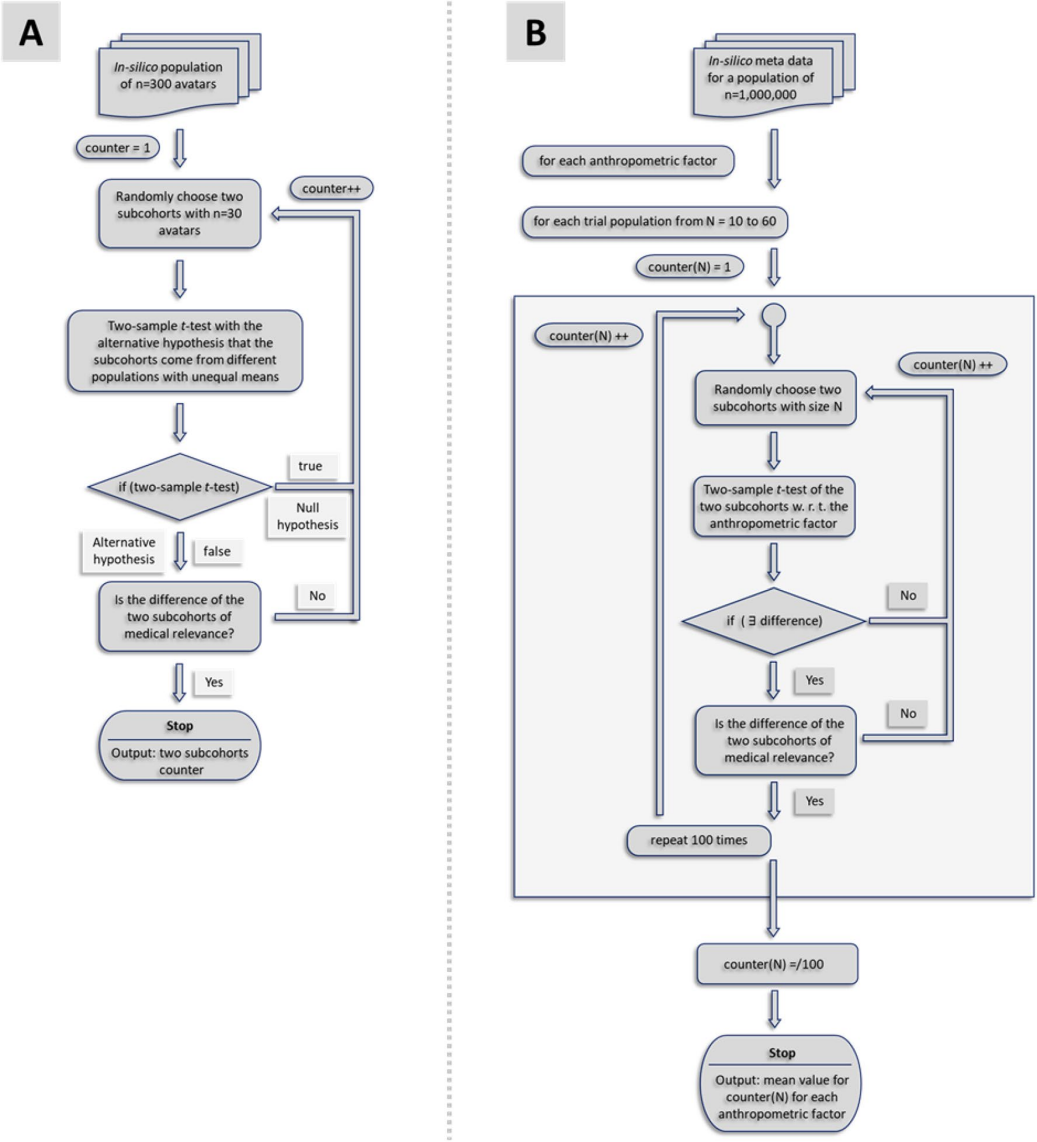
(**A**) Flowchart for the generation of two subcohorts with a clinically relevant difference in anthropometric characteristics based on the in silico population of the 300 generated avatars. (**B**) Flowchart illustrating the algorithm to compute the mean number of iterations to generate two subcohorts with a medical relevant difference for a specific anthropometric parameter for trial population sizes from 10 to 60 one hundred times based on the meta data of a population of 1,000,000 subjects.

From the population of avatars (n = 300) in silico trial cohorts with n = 30 avatars are generated (Algorithm A, Fig. 2) until two populations are found, which are significantly different (two-sample *t*-test, p = 0.05) with a statistical difference expressed by the effect size considered clinically relevant as explained below. The two differing cohorts are then analysed with respect to their gait simulation results whether statistically significant differences at the macro-scale, i.e. the cohort, propagate downstream to the fracture gap, thereby describing the impact of anthropometric variation on fracture gap micromechanics, giving an impression of the possible impact on fracture trial outcome.

### Probability to generate two, with respect to anthropometric parameters statistically different trial cohorts, given the same power assumptions

**Figure 3:**
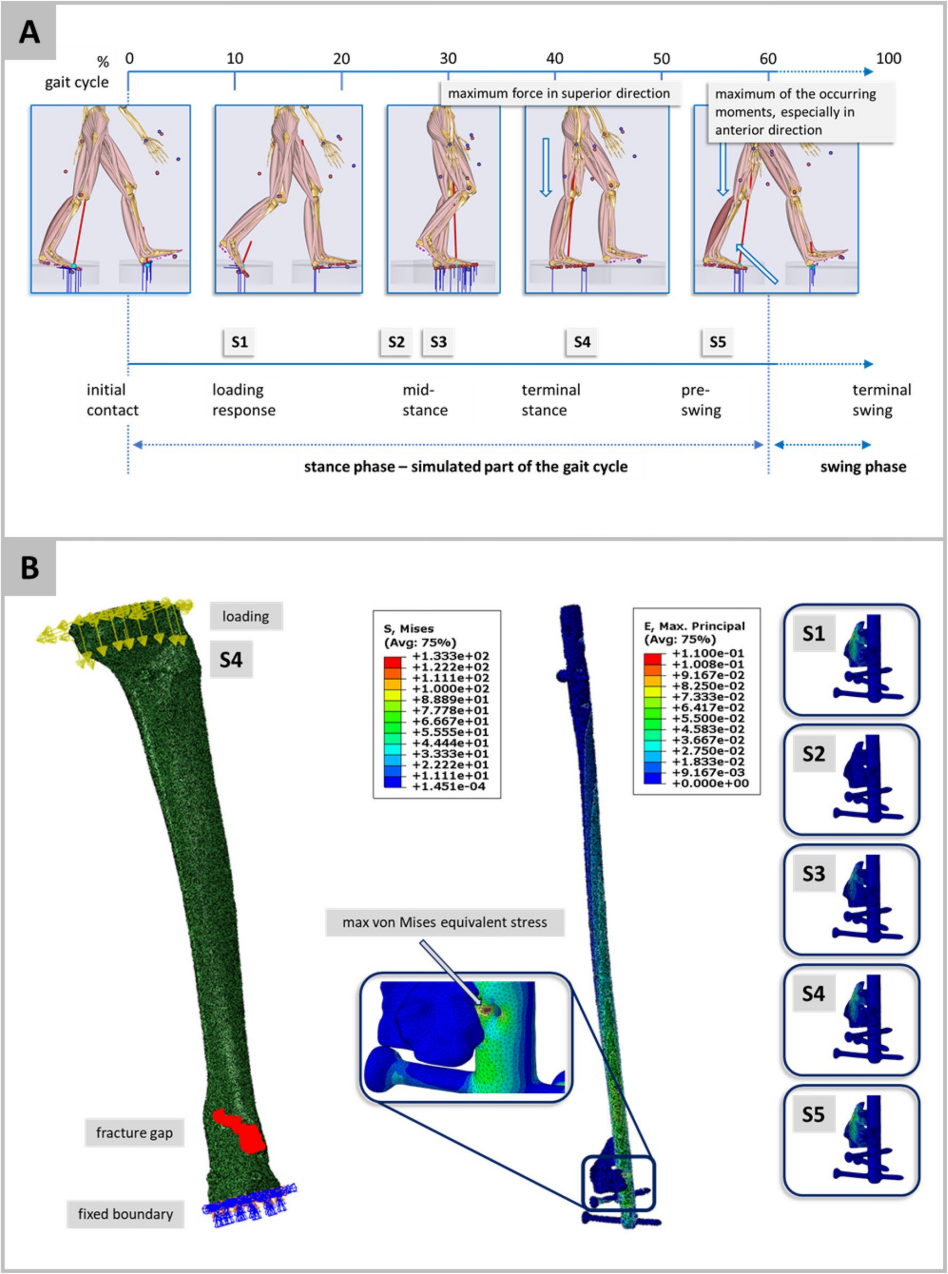
(**A**) Graphical placement of the five simulation points in the gait cycle. The shown musculoskeletal simulation images are performed in AnyBody (AnyBody Technology A/S, Aalborg, Denmark) on patient motion capturing data to illustrate a gait cycle. The highest correlation coefficients for the body weight occur in point S4 where the maximum force in the superior direction acts. For the other anthropometric factors BMI, body height and tibia length, the highest correlation coefficients appear in point S5 where the maximum moments occur during the step forward. (**B**) Typical result of the simulation process; on the left, the set-up of the boundary conditions is shown for the force maximum of the superior direction (S4) for one avatar model; in the middle, the von Mises equivalent stress distribution of the implant is shown for this simulation, and on the right, the maximum principle strain in the fracture gap is shown for this avatar and all five simulations at the landmarks. The influence of the gait dynamics on the fracture gap can be clearly seen.

The issue of trial cohort sizes and the influence of anthropometric factors on the statistical equivalence of cohorts of same size, is analysed by means of a second in silico population (n = 1,000,000) which exclusively refers to the metadata (age class, BMI, body height, body weight, tibial length). Algorithm B (Fig. 2) maps the recruitment scenario of a clinical trial to the in silico context. For trial cohorts ranging from 10 to 60 subjects, pairs of cohorts are randomly drawn from this significantly larger population. This is repeated until a pair of trial cohorts proofs a statistically significant difference (two-sample t-test, p = 0.05) which is considered to be clinically relevant. To quantify the relevance of statistically significant differences Cohen’s d is calculated for values between d = 0.5 (medium effect size) and d = 0.9 (large effect size) (Algorithm B, Fig. 3). This process is performed for all four anthropometric factors considered. To reduce the influence of statistical outliers, the algorithmic procedure was repeated 100 times each and the mean number of iterations was determined for each trial size from 10 to 60 subjects.

## Results

### Correlation between anthropometric parameters and the mechanical stimulus in the fracture gap

Based on 1500 biomechanical FE simulations of n = 300 avatars at the landmarks S1 to S5 of the stance phase, Fig. 3A illustrates the results of avatar 16, with the virtual anthropometric meta data: age group 5 (40–45 years old), BMI 25·22, body weight 81·54 kg, body height 179·7 cm and a tibial length of 40.39 cm.

Correlation (p = 0.01, two-sided) for median values of the mechanical relevant quantities in fracture gap and callus area and the anthropometric parameters is significant, with the correlation coefficients varying between very strong correlations to less pronounced relationships (Table 1). For BMI, body height and tibia length, the highest correlation coefficients occur for the hydrostatic strain at S5. For body weight, many correlation coefficients are above 0.90, especially at S4. In all cases, the results regarding the landmarks are clearly mechanically determined. For the body weight, its influence is highest, if the force maximum is reached in the axial direction. Similarly, the highest influence for the remaining three anthropometric factors, i.e. highest correlation coefficients, occurs when the moments acting in the distal tibia reach their maximum at landmark S5, cf. Fig. 3A.

**Table 1:** Correlation coefficients from a two-sided Pearson test between the anthropometric factors during the five identified landmarks in the gait cycle and the median values for the mechanical quantities hydrostatic strain, octahedral shear strain, maximum principal strain and J2 (second invariant of the deviatoric strain tensor) for the fracture gap and the callus area.

	Anthropometric Parameter	Fracture gap					Callus area		
		Hydrostatic strain	Octahedral shear strain	Max principal strain	J2	Hydrostatic strain	Octahedral shear strain	Max principal strain	J2
S1	Body weight	− 0.987**	0.987**	0.984**	− 0.974**	0.983**	0.984**	0.986**	− 0.977**
	Body height	− 0.612**	0.610**	0.626**	− 0.624**	0.619**	0.628**	0.609**	− 0.608**
	BMI	− 0.795**	0.797**	0.786**	− 0.768**	0.786**	0.783**	0.798**	− 0.781**
	Tibia Length	− 0.614**	0.613**	0.627**	− 0.626**	0.622**	0.629**	0.611**	− 0.610**
S2	Body weight	− 0.989**	0.987**	0.984**	− 0.972**	0.984**	0.983**	0.983**	− 0.976**
	Body height	− 0.587**	0.606**	0.629**	− 0.630**	0.626**	0.634**	0.620**	− 0.603**
	BMI	− 0.813**	0.799**	0.783**	− 0.762**	0.784**	0.778**	0.788**	− 0.782**
	Tibia Length	− 0.589**	0.608**	0.631**	− 0.632**	0.628**	0.636**	0.622**	− 0.606**
S3	Body weight	− 0.993**	0.990**	0.946**	− 0.967**	0.984**	0.986**	0.946**	− 0.971**
	Body height	− 0.586**	0.604**	0.589**	− 0.617**	0.626**	0.633**	0.579**	− 0.590**
	BMI	− 0.818**	0.804**	0.762**	− 0.764**	0.784**	0.783**	0.768**	− 0.784**
	Tibia Length	− 0.588**	0.607**	0.590**	− 0.618**	0.629**	0.635**	0.581**	− 0.592**
S4	Body weight	− *0*.994**	0.992**	0.989**	− 0.981**	0.988**	0.990**	0.991**	− 0.983**
	Body height	− 0.563**	0.561**	0.580**	− 0.579**	0.579**	0.581**	0.554**	− 0.560**
	BMI	− 0.829**	0.831**	0.818**	− 0.802**	0.816**	0.817**	0.835**	− 0.816**
	Tibia Length	− 0.564**	0.562**	0.581**	− 0.579**	0.580**	0.581**	0.554**	− 0.561**
S5	Body weight	− 0.777**	0.992**	0.895**	− 0.925**	− 0.708**	0.961**	0.892**	− 0.953**
	Body height	− 0.492**	0.542**	0.650**	− 0.702**	− 0.913**	0.715**	0.574**	− 0.541**
	BMI	− 0.956**	0.841**	0.666**	− 0.663**	− 0.481**	0.703**	0.707**	− 0.791**
	Tibia Length	− 0.492**	0.544**	0.652**	− 0.707**	− 0.928**	0.720**	0.575**	− 0.542**

The respective maximum values are highlighted in italics. To perform the correlation analysis, for each simulation, the strain quantities in the fracture gap and in the callus area for each finite element, i.e. the corresponding C3D10 tetrahedron, were evaluated. Then, statistical quantities (e.g. median as shown in this table) were evaluated for the corresponding vectors in which these strain quantities are stored and transferred to the correlation test.

**The correlation is significant at the 0.01 level (2-sided).

**Figure 4:**
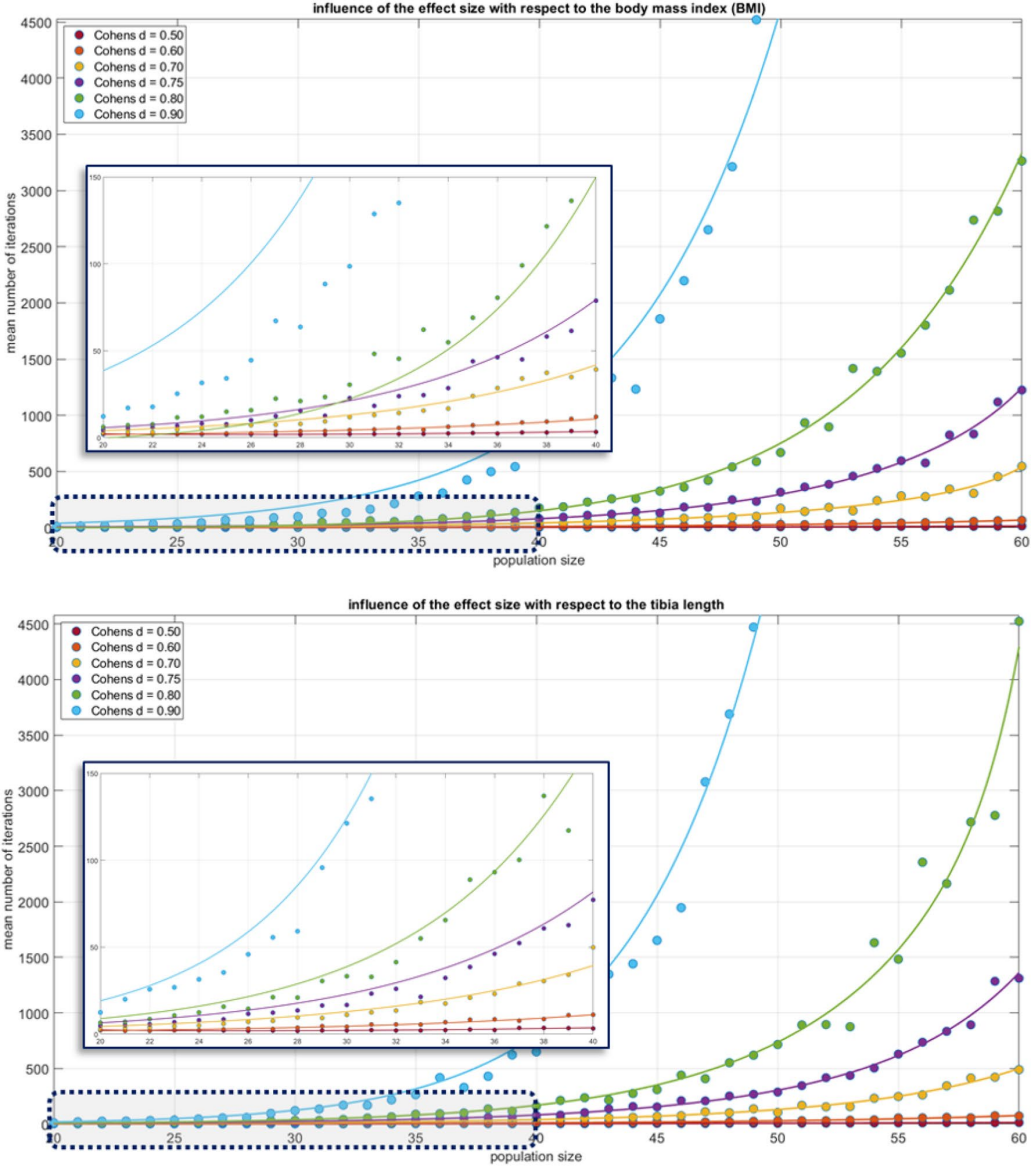
Result of the process shown in flowchart B, Fig. 2, for the BMI. For the other anthropometric factors, the results are similar.

The change in mechanical loading at the transition from S4 to S5 explains the change in sign of the correlation coefficients of hydrostatic strain in the case of the posteriorly located callus area. The mechanical stimulus changes in a relevant number of mesh cells from compression to tension. Interestingly, in the anterior region of the fracture most mesh cells are subjected to tensile forces throughout the whole gait cycle. The strain quantities based on shear (octahedral shear strain, J2), representing the distortion, correlate most strongly with the body weight. However, for body height and tibia length, all correlation coefficients are above 0.50, still indicating a clear linear relationship (Fig. 4).

**Figure 5:**
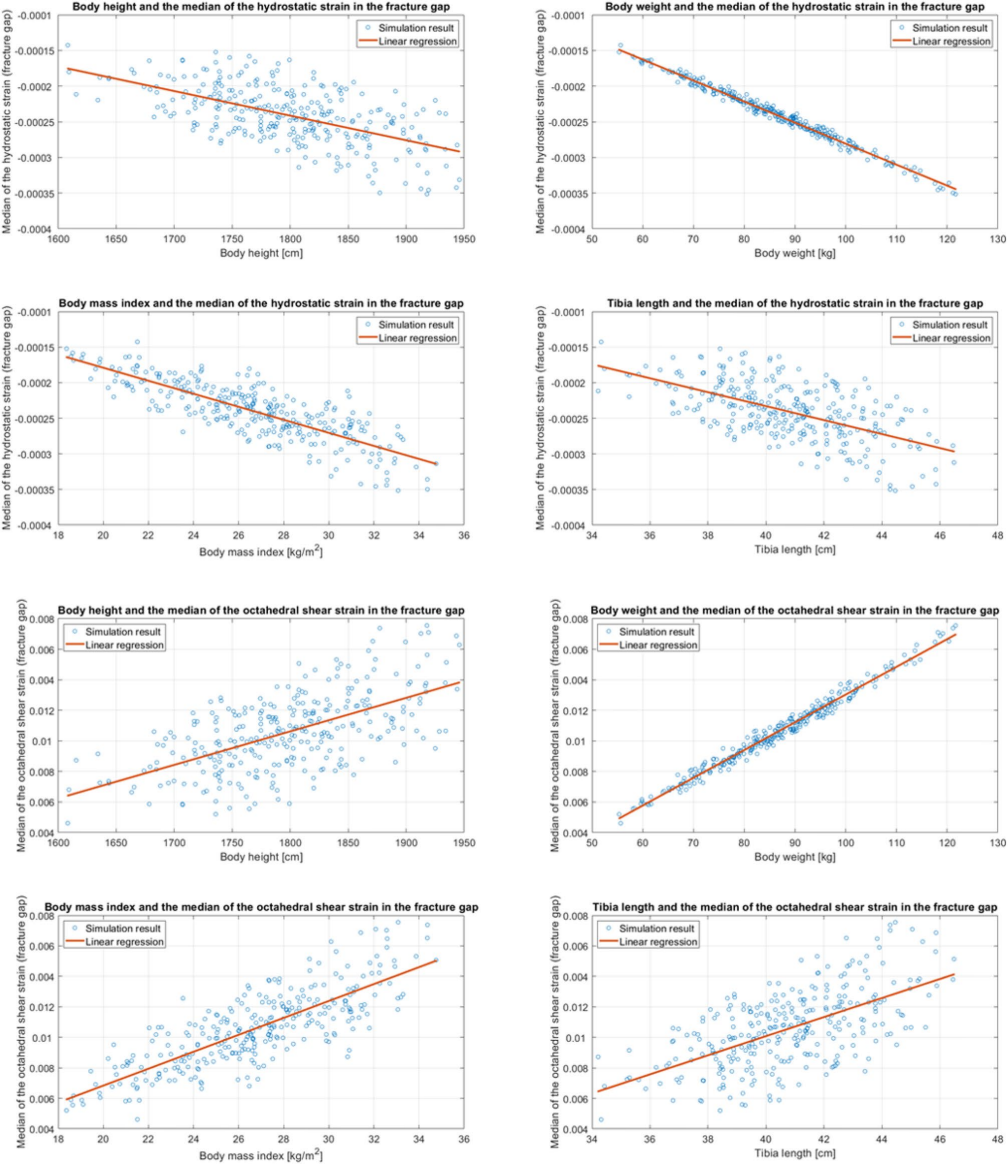
Linear Regression between the four anthropometric parameters and the two mechanical quantities hydrostatic strain and octahedral shear strain in the fracture gap. The plots and the associated linear regression analysis are derived from the data encapsulated in Table 1. Notably, there's an evident trend concerning body weight. This observation aligns with expectations, as body weight, when incorporated as a boundary condition in the simulations, has a pronounced impact on the results.

Figure 5 also elucidate the relationship between the anthropometric parameters and strain quantities identified for influencing fracture healing. Notably, as body weight rises, both hydrostatic and octahedral shear strains demonstrate a pronounced increase. Conversely, an augmented BMI indicates decreased hydrostatic strain but increased octahedral shear strain. These insights underscore the significance of understanding how individual anthropometric metrics influence interfragmentary movement, which is paramount for comprehending fracture mechanics and healing trajectories in clinical scenarios.

### *Influence of anthropometric parameters on significant statistical difference of randomly composed *in silico* trial cohorts*

Table 2 shows a result where the algorithm reaches an effect size of Cohen’s d = 1·21 (the if-query checking for medical relevance in flowchart A, Fig. 2, searches for Cohen’s d values greater than or equal to 0.9) after just 4 iteration cycles. Here, we also have the possibility to evaluate the respective simulation results at the five landmarks of the gait cycle, cf. Table 2. Regardless the difference in the anthropometric factors in both cohorts, they obviously correlate in nearly all cases with the relevant quantities for fracture healing. Thus, when anthropometric parameters vary over a range representing the anthropological measures of the general population, even in the case of a single fracture morphology with the same osteosynthesis, trial populations may occur that are both statistically and clinically relevantly different, but still provide similar correlations for fracture gap mechanics.

**Table 2:** Correlation coefficients from a two-sided Pearson test between the anthropometric factors of during the five identified landmarks of the gait cycle and the median values for the mechanical quantities hydrostatic strain and octahedral shear strain for the fracture gap and the callus area for both cohorts with a relevant difference.

Cohort	Landmark	Anthropometric factor	Fracture gap		Callus area	
			Hydrostatic strain	Octahedral shear strain	Hydrostatic strain	Octahedral shear strain
1	S1	Body weight	− 0.9866**	0.9876**	0.9772**	0.9828**
		Body height	− 0.7108**	0.7064**	0.7086**	0.7264**
		BMI	− 0.7221**	0.7266**	0.7109**	0.7067**
		Tibia Length	− 0.7086**	0.7056**	0.6973**	0.7217**
2		Body weight	− 0.9307**	0.9927**	0.9788**	0.9518**
		Body height	− 0.5873**	0.6621**	0.5590**	0.5891**
		BMI	− 0.6748**	0.7658**	0.7423**	0.6971**
		Tibia Length	− 0.6211**	0.6586**	0.5938**	0.6243**
1	S2	Body weight	− 0.9922**	0.9904**	0.9817**	0.9869**
		Body height	− 0.5798**	0.5937**	0.6223**	0.6155**
		BMI	− 0.8000**	0.7898**	0.7612**	0.7723**
		Tibia Length	− 0.5972**	0.6116**	0.6404**	0.6312**
2		Body weight	− 0.9894**	0.9858**	0.9824**	0.9808**
		Body height	− 0.5862**	0.6106**	0.6175**	0.6357**
		BMI	− 0.8029**	0.7848**	0.7751**	0.7640**
		Tibia Length	− 0.0619**	0.6430**	0.6520**	0.6693**
1	S3	Body weight	− 0.9891**	0.9878**	0.9798**	0.9816**
		Body height	− 0.6889**	0.7018**	0.7142**	0.7324**
		BMI	− 0.7415**	0.7301**	0.7107**	0.7005**
		Tibia Length	− 0.6853**	0.7003**	0.7054**	0.7279**
2		Body weight	− 0.9383**	0.9280**	0.9800**	0.9352**
		Body height	− 0.5651**	0.5895**	0.5700**	0.5996**
		BMI	− 0.6965**	0.6709**	0.7375**	0.6723**
		Tibia Length	− 0.5978**	0.6215**	0.6052**	0.6340**
1	S4	Body weight	− 0.9919**	0.9948**	0.9799**	0.9879**
		Body height	− 0.6672**	0.7831**	0.6707**	0.6847**
		BMI	− 0.7610**	0.7954**	0.7425**	0.7443**
		Tibia Length	− 0.6628**	0.6586**	0.6568**	0.6774**
2		Body weight	− 0.9369**	0.9465**	0.9836**	0.9541**
		Body height	− 0.5369**	0.5444**	0.5153**	0.5398**
		BMI	− 0.7128**	0.7187**	0.7747**	0.7302**
		Tibia Length	− 0.5706**	0.5776**	0.5501**	0.5748**
1	S5	Body weight	− 0.7417**	0.9393**	− 0.8601**	0.9720**
		Body height	− 0.1744	0.5948**	− 0.9589**	0.8739**
		BMI	− 0.9412**	0.6796**	− 0.4467**	0.6846**
		Tibia Length	− 0.1360	0.6279**	− 0.9495**	0.8549**
2		Body weight	− 0.7957**	0.9969**	− 0.7829**	0.9721**
		Body height	− 0.1349	0.6647**	− 0.9286**	0.7963**
		BMI	− 0.9327**	0.8921**	− 0.4743**	0.7872**
		Tibia Length	− 0.0652	0.6258**	− 0.9367**	0.7699**

Only the four highlighted cases show no relevant correlation.

**The correlation is significant at the 0.01 level (2-sided).

### Probability to generate two, with respect to anthropometric parameters statistically different trial cohorts given identical power assumptions

Based on the hypothesis that anthropometric factors have an influence on fracture healing, the goal of the algorithm B, Fig. 2, is to assess at which trial sizes this becomes relevant. Figure 4 shows the results of algorithm B for the BMI and the tibia length as anthropometric measures. For all values of Cohen’s d, the output mean number of iterations increases exponentially, beginning, depending on the effect size, between the population sizes n = 30 and n = 60. Based on these results, we can say that for a collective of n = 50 patients and an effect size with Cohen´s d = 0.9, an average of almost 5000 iterations were necessary to generate our requested difference and this will therefore not occur in clinical practice. However, for smaller and lower values of Cohen´s d, the average number of iterations is only about 10 and also for a Cohen’s d = 0.8 only about 30 iterations were needed on average.

## Discussion

Well defined micromechanical strain in the fracture gap is the necessary condition for fracture healing^8,9,18,19^. The actual osteogenic effect of the mechanical impact is modulated by host factors, biological factors, and iatrogenically, i.e. by the type and skill the osteosynthesis is performed. Micromechanics in the fracture gap is defined by the fracture pattern, the configuration of the osteosynthesis, and hypothetically by anthropometric factors. Taking advantage of an in silico approach, which allows to systematically vary anthropometric parameters, while fracture pattern and osteosynthesis remain unchanged, this in silico study investigated the impact of anthropometric variables on fracture gap micromechanics. Fracture morphology and osteosynthesis are given conditions, while mechanical effects exerted by anthropometric parameters are dynamic variables as they depend on the patient's level of activity and compliance thereby defining over-, under- or physiological impact in the fracture gap. So far there are neither rules to systematically integrate these factors into clinical decision making and rehabilitation, nor are there rules regarding the impact of anthropometric parameters on trial population composition in fracture trials.

Generally, the hypothesis that anthropometric parameters correlate with mechanical quantities in the fracture gap is confirmed (Table 1). The fact that the body weight defines the absolute amount of force acting in the fracture gap is straightforward and trivial. The more interesting observation is, that the tibial length achieves an r^2^ ranging from 0.24 to 0.49, depending on the type of strain and the phase of the gait cycle (Fig. 3A). The magnitude of the effect is too small to generally determine the mechanical conditions in the fracture gap, but it is large enough to reduce the number of subjects of the trial cohort that hit the window of optimal mechanical stimulus^18,19^ by an unknown number, thereby conflicting with the integrity of the power calculation. Thus, the issue of anthropometric variables is obviously more than a methodological meta debate but rather a confounder that might be strong enough to interfere with the idea that in RCT differences like these are levelled out by the principle of randomization. Looking for a variable like tibial length in the cohorts of a randomized trial might reveal that this variable is evenly distributed, thus ostensibly the levelling effect of randomization appears successful. Most importantly, this study addresses the meso-scale characteristic of the fracture gap, i.e. the vicinity where mechanoinduction of fracture healing actually takes place, as endpoint. Mechanically the fracture gap is the place where the mechanical effects of several macro-scale variables (body weight, body height, tibia length, implant, compliance) become a joint input signal for the complex cellular interactions that ultimately result in bone formation, i.e. fracture healing.

To better understand the effect of omitted anthropometric variables on trial cohort design, a recruitment process was retraced virtually. The fact that the virtual trial cohort contains only a single, always identical, fracture type is a methodological artifice to facilitate the computational processes. As the endpoint of this investigation is not healing of the model fracture at the macro-scale level, but rather the mechanobiological loading at the meso-scale level (inside the fracture gap) this facilitation does not reduce the meaningfulness of the results. Resulting from the oblique orientation of any fracture gap each voxel or tetrahedral mesh cell has its own mechanobiological profile, thus the limitation to a single fracture type at the macro-scale does not necessarily curtail the information on the meso-scale level. As a given BMI can be achieved with different combinations of body height and body weight, different tibia measures, with distinct modulating effects on meso-scale mechanobiology, have to expected. Retracing trial recruitment showed that the risk of generating statistically different trial cohorts with respect to anthropometric variables decreases exponentially with larger cohorts, which is to be expected. However, given the assumption that a cut off of 50 iterations is a relevant dimension in clinical research, i.e. that one in fifty cohorts is different regarding anthropometric variables, trials with cohorts larger than 40 participants can achieve effect sizes of more than 0.7. Thus, larger trial populations appear to be safe from random bias by uncontrolled anthropometric parameters. However, for smaller group sizes and effect sizes between medium and large, the opposite is the case (Fig. 4). Based on these observations there seems to be no fundamental methodological limitation regarding the implementation of randomized, controlled fracture trials in general.

However, an analysis of tibia fracture trials listed in *clinicaltrials.gov* shows a total of 1963 trials relating to tibia fractures, while only 28 explicitly name fracture healing as endpoint. Among those only 15 address surgical fracture care, i.e. osteosynthesis. Only one trial was actually terminated and published, while the remaining trials were not published, or did not reach the targeted enrolment numbers. Together with the observations reported in this study, it becomes clear that randomized controlled trials relating to the necessary endpoint of fracture care, i.e. fracture healing, are caught in a dilemma as either they require enrolment numbers which are difficult to achieve in clinical reality, or uncontrolled anthropometric parameters, as demonstrated in the present simulation study, introduce uncontrolled bias regarding the interpretation of the trial.

## Conclusion

The present analysis ends up in a paradox. From the perspective of the individuum, anthropometric parameters do have a relevant impact on fracture gap mechanics. In terms of trial cohorts this effect can be compensated for by increasing the size of trial cohorts, i.e. power calculation of RCTs works.

The discussion on whether randomised trials are the tools of choice to generate clinically meaningful evidence in orthopaedic trauma surgery is long-standing, as there are only few fracture RCTs, with an even lesser number successfully recruiting the scheduled number of patients, and many never getting published. Notably, the implementation of supportive infrastructure, e.g. centres for clinical studies, in many places, did not increase the number of successful fracture RCTs.

Given the fact that in this analysis, for proof of concept purposes, a single fracture was used, the size of trial cohorts naturally increases in a real-world trial with fracture patters showing morphological variability, not to speak about the systematic implementation of anthropometric variables. The achievement of adequate sample sizes and hence statistical power is a notorious issue in real world fracture trials. Farrow et al. report on 25 orthopaedic trials published in high impact journals. More than half of these did not meet the estimated sample size for the primary outcome criterion, and only 56% of these studies provided adequate justification for the minimum clinically important difference (MCID) in the population assessed.

With simulation approaches based on routine computed tomography data of osteosynthetically treated fractures coming within reach for clinical routine application, a new type of clinical data becomes available. The observations reported in this study provide insight into the mechanical conditions in the space where mechanobiology is translated to fracture healing, i.e. the fracture gap.

Given these considerations, a new perspective on fractures becomes possible. A meso-scale focused description of a fracture would then understand a fracture as an aggregation of voxels which are subjected to different mechanical loading scenarios depending on the geometry of the fracture, anthropometric measures, the osteosynthesis and patient behaviour. Each voxel that experiences a mechanical loading within the mechanobiological optimal window has the option to proceed to bone formation given the modulating biological factors permitting this.

This new perspective, i.e. thinking fracture care from the fracture gap, paves the way for a fracture classification that focuses on the mechanobiological conditions in the fracture gap, rather than on the preoperative morphology of the fracture. Future research will show whether this results in a restructuring of fracture classification, an additional fracture classification, a tool to guide postoperative weight bearing, or a tool to categorise surgical performance. Apart from applications in clinical medicine the approach developed here might also offer the opportunity to conduct virtual trials to facilitate the financial and ethical aspects of implant development.

### Supplementary Information

Supplementary Information.

## Data Availability

No supplementary data is available. The original data presented in the study are included in the article, further inquiries can be directed to the corresponding author/s.
