# Supplementary material for: Reappraisal of clinical trauma trials: the critical impact of anthropometric parameters on fracture gap micro-mechanics—observations from a simulation-based study
Source: Sci Rep. 2023 Nov 22;13:20450. doi: 10.1038/s41598-023-47910-2 (PMC10665421; doi:10.1038/s41598-023-47910-2)
Supplement: Supplementary file 1 — Supplementary Information. [file 41598_2023_47910_MOESM1_ESM.docx]

Throughout the manuscript, standard definitions from continuum mechanics are used. In order to analyze the simulation results from the Abaqus computations made with respect to the strain quantities commonly used in the literature on the mechanics of bone healing, the following steps were undertaken:

- Export of the local symmetric strain tensor, given by the following definition,

$\boldsymbol{\varepsilon}= \left( \begin{matrix} \varepsilon_{xx} & \varepsilon_{xy} & \varepsilon_{xz} \\ \varepsilon_{xy} & \varepsilon_{yy} & \varepsilon_{yz} \\ \varepsilon_{xz} & \varepsilon_{yz} & \varepsilon_{zz} \end{matrix} \right)$,

for every integration point of each tetrahedral element of the fracture gap and the callus area. The export was realized with a script in Abaqus python resulting in a csv (comma separated values) file with the following data format per line:

element number, local integration point number, $\varepsilon_{xx}, \varepsilon_{yy},\varepsilon_{zz},\varepsilon_{xy},\varepsilon_{xz},\varepsilon_{yz}$

- The data was then loaded into Matlab for further processing.
- Based on the csv files (one strain data file for every simulation) the strain invariants were computed in a Matlab routine:

$$\begin{matrix} I_{1} & = & \mathrm{tr}(\boldsymbol{\varepsilon}) \\ I_{2} & = & \frac{1}{2}\left( \left[ \mathrm{tr}\left( \boldsymbol{\varepsilon} \right) \right]^{2}-\mathrm{tr}(\boldsymbol{\varepsilon}^{2}) \right) \\ I_{3} & = & \det\left( \boldsymbol{\varepsilon} \right) \end{matrix}$$

where $\mathrm{tr}(\boldsymbol{\varepsilon})$ is the trace of the strain tensor.

- In addition to the three invariants, the principal strains $\varepsilon_{1},\varepsilon_{2},\varepsilon_{3}$ were computed via an eigenvalue decomposition with the appropriate Matlab functions.
- In the next step, the hydrostatic strain was computed for every given strain tensor via the formula:

$$hydrostatic strain=\frac{1}{3}(\varepsilon_{1}+\varepsilon_{2}+\varepsilon_{3})$$

- Then, the octahedral shear strain was computed with the following formula:

$$octahedral shear strain= \left( \frac{2}{3} \right)\sqrt{\left( \varepsilon_{1}-\varepsilon_{2} \right)^{2}+\left( \varepsilon_{2}-\varepsilon_{3} \right)^{2}+\left( \varepsilon_{3}-\varepsilon_{1} \right)^{2}}$$

- In order to compute J2, i.e., the second invariant of the deviatoric strain tensor, the deviatoric strain tensor was computed by subtracting the mean strain tensor from the strain tensor:

Mean strain: $\varepsilon_{M}=\frac{1}{3}I_{1}$

Deviatoric strain tensor = $\left( \begin{matrix} \varepsilon_{xx}-\varepsilon_{M} & \varepsilon_{xy} & \varepsilon_{xz} \\ \varepsilon_{xy} & \varepsilon_{yy}-\varepsilon_{M} & \varepsilon_{yz} \\ \varepsilon_{xz} & \varepsilon_{yz} & \varepsilon_{zz}-\varepsilon_{M} \end{matrix} \right)$

- With an eigenvalue decomposition of the deviatoric strain tensor, the deviatoric principal strains $\varepsilon_{1}^{'}, \varepsilon_{2}^{'}, \varepsilon_{3}^{'}$are calculated and used to compute J2:

$$J2= \varepsilon_{1}^{'}*\varepsilon_{2}^{'}+\varepsilon_{2}^{'}*\varepsilon_{3}^{'}+\varepsilon_{1}^{'}*\varepsilon_{3}^{'}$$
